# A rare etiology of infantile dyspnea: Oropharyngeal teratoma

**DOI:** 10.1016/j.ijscr.2024.110062

**Published:** 2024-07-20

**Authors:** Chiboub Dorra, Romdhane Nadia, Jouini Selima, Cherif Maya, Chedly Achraf, Mbarek Chiraz

**Affiliations:** a ENT Department, Habib Thameur Hospital, Tunis, Tunisia; b Cytopathology Department, Habib Thameur Hospital, Tunis, Tunisia

**Keywords:** Infant, Teratoma, Oropharyngeal lesion, Dyspnea

## Abstract

**Introduction and importance:**

Oropharyngeal teratoma is a rare congenital tumor that grows slowly and can be suspected prenatally. It entails the obstruction of upper airway and upper digestive tract. Clinical examination is always completed by imaging particularly the MRI.

**Case presentation:**

We present a case of an eight- month -old infant who presented an oropharyngeal mass which caused intermittent dyspnea and feeding difficulties.

**Discussion:**

The treatment is only surgical and histopathological examination confirms the diagnosis.

**Conclusion:**

Early diagnosis is key to successful management of oropharyngeal teratomas.

## Highlights

• Oropharyngeal teratomas are rare congenital tumors.
• Diagnosis is based on CT and MRI.
• Early management is essential.
• Treatment is surgical.

## 1 Introduction

Teratoma is the most common infant neuronal congenital tumor, it represents 25 % of all benign and malignant tumors [1]. It usually develops in the sacrococcygeal region and only 6 to 10 % are located in the head and neck region, with a male female ratio from 1:6 to 1:3 [[1], [2], [3]]. It contains differentiated tissues foreign to the region in which it develops. The risk for head and neck localization is a partial or complete obliteration of the upper airway. The diagnosis can be made during the prenatal period thanks to recent advances in prenatal magnetic resonance imaging (MRI) [4] that allows to plan the surgical management of the newborn depending on the characteristics of the lesion. The work has been reported in line with the SCARE criteria [5].

## 2 Presentation of case

**Fig. 1:**
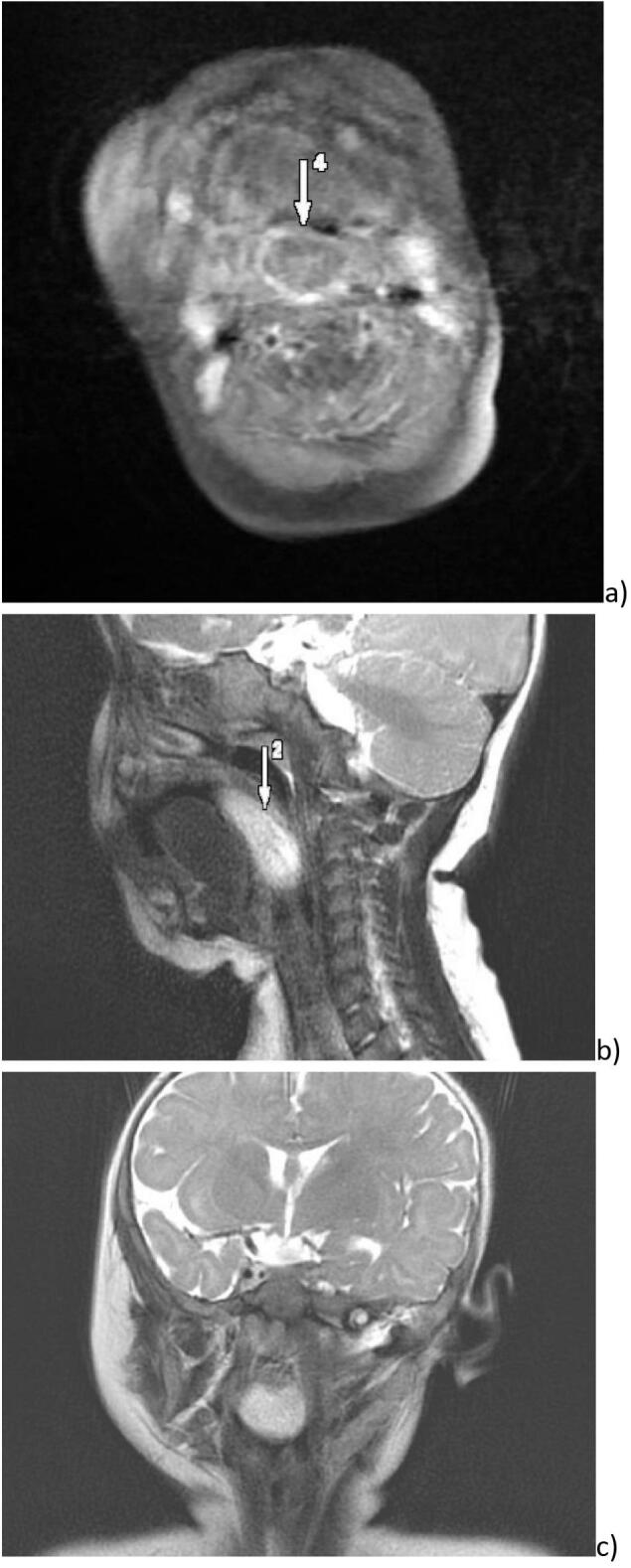
Preoperative MRI in different sections a) axial, b) sagittal and c) coronal showing a well limited tissular lesion in hypersignal in the TWO sequences, this mass is filling the oropharynx.

We report a case of an eight-month-old female infant who presented to our ENT emergencies with intermittent respiratory distress and difficulties to be fed. The symptoms were evolving for 2 months while she started to lose weight. On the first examination, there was no respiratory distress, there wasn't any cervical node, and systemic examination was normal. The oropharyngeal and endoscopic examination showed a voluminous protruding mass over the tongue, coming from the left pharyngeal wall. Otoscopy did not show any abnormalities. A Magnetic resonance imaging (MRI) was performed and showed an oropharyngeal tissular mass, measuring 51 ∗ 26 ∗ 14 mm (Fig. 1). It seemed to come from the base of the tongue. The mass was lobed, homogeneous, with a high intensity signal in T1 and T2 sequencies. The injection of gadolinium showed that it was vascularized.

**Fig. 2:**
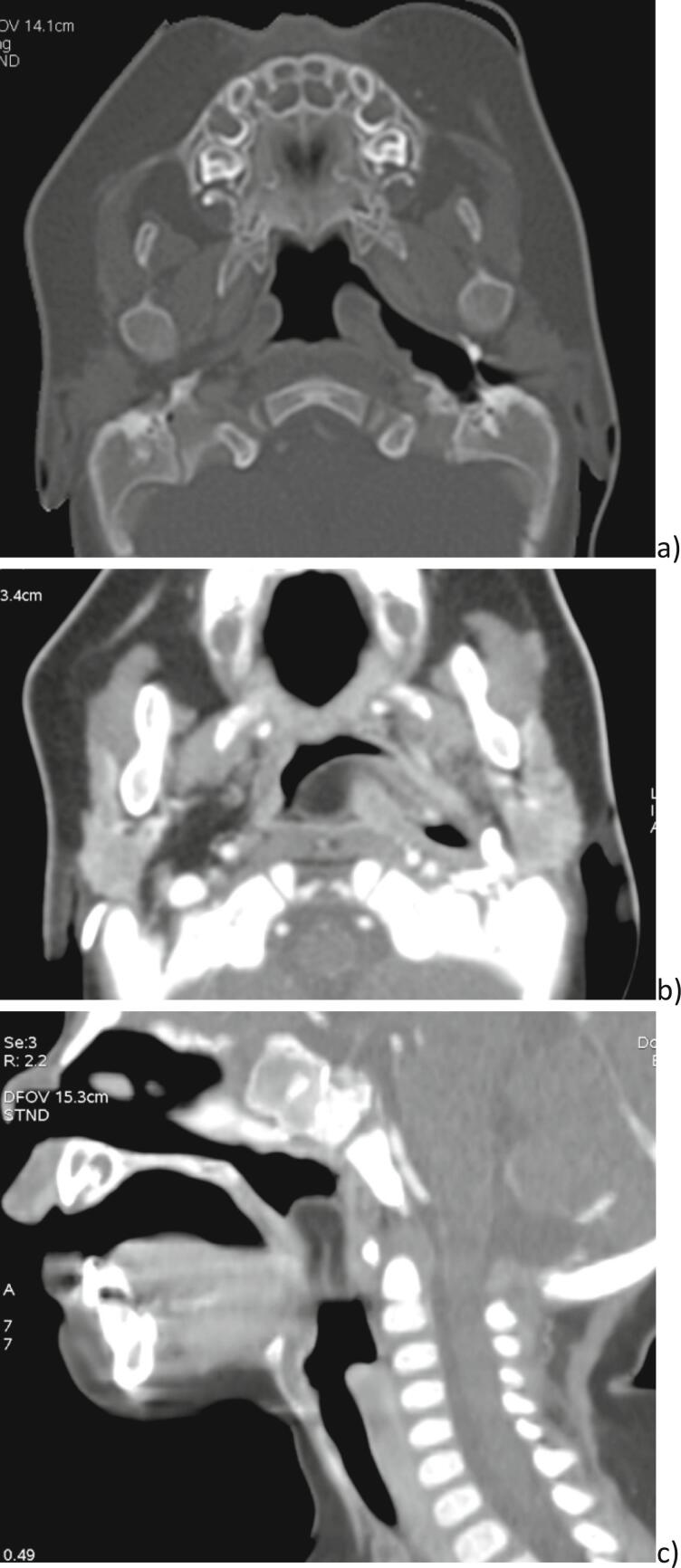
Preoperative tomodensitometry aspect showing: a) a dilation of the left eutachian tube but without bone damage, b) a protrusing tissular mass well limited pediculated in the left eustachian tube where it is inserted, c) the tissular lesion filling the aerodigestive tract.

We completed the examination with a Computed Tomography (CT-scan) that objectified a well-defined mass in the left parapharyngeal space measuring 30 ∗ 17 ∗ 8 mm. The lesion had a fat tissular CT density. The site of attachment was the left Eustachian tube. The mass was oblong, lying on the tongue. It spread in the hypopharyngeal space pushing in front the epiglottis and arriving in contact with the left arytenoid (Fig. 2). Tympanometry was normal.

The treatment was strictly surgical with Rose's position, under complete anesthesia. Intraoperatively, we found a pale white pedunculated mass, measuring 3.5 cm, originating from the left lateral parapharyngeal wall. We completely resected the tumor along with its site of insertion through a transoral approach. A subsequent primary closure of the palatal defect was made.

The intervention proceeded without any complications.

**Fig. 3:**
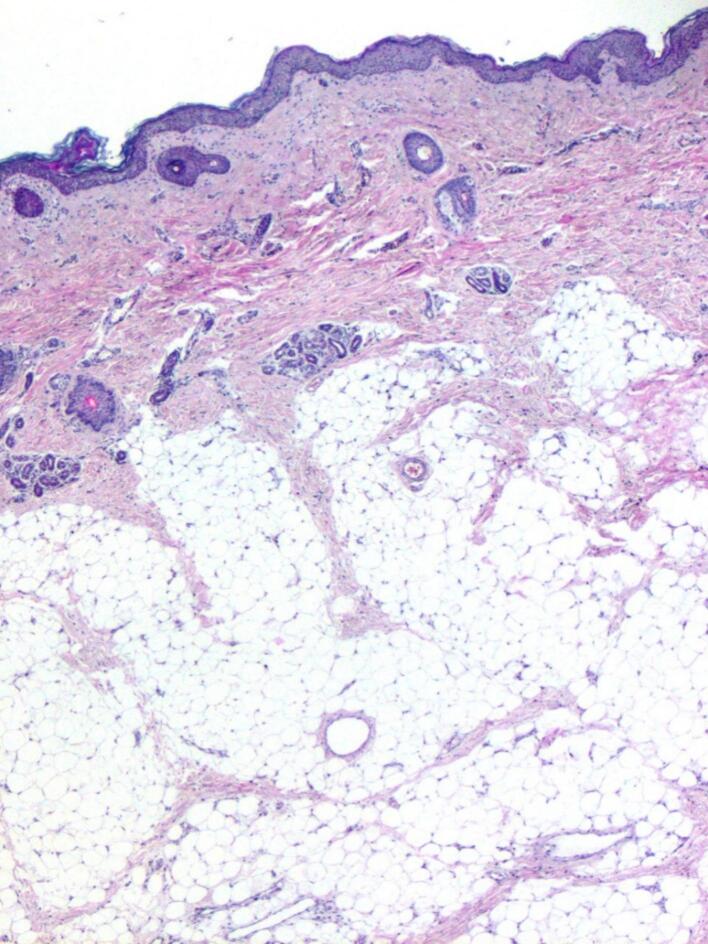
Histological aspect post operatively showing a polypoid mass covered by squamous epithelium with pilosebaceous units. Under this epithelium there was mainly adipose tissue (HE, ×100).

Histopathological examination showed tissues derived from epidermal and mesodermal germ layers. The epidermal component comprised a squamous epithelium with pilosebaceous units covering the surface of the polypoid formation. The mesodermal derivate were predominantly adipose tissue, muscle, and cartilage. There was no evidence of immature neural elements. Overall, it was suggestive of a mature oropharyngeal teratoma (Fig. 3).

No complications such as infection or bleeding were reported following surgery. The infant was well breastfed, and her weight-loss curve improved. The MRI performed 6 months after surgery was normal and the patient didn't show signs of recurrence after a 4-year follow-up.

## 3 Discussion

Teratoma contains at least 2 of the three germinal layers that are ectoderm, mesoderm and endoderm. There are three types of teratoma: benign (contain mature tissues), mixed (immature tissue) and malignant. The last one represents a small quota [4]. However, even if they are benign, teratomas a can have serious repercussions and be life threatening such as in our case if the tumor had grown bigger. Oropharyngeal teratoma (also named epignathus) can present several symptoms depending on the size and the location, from all degrees of dyspnea, to feeding difficulties or just a mass discovered by the parents [4,6,7]. In our case, the patient didn't feed properly. In front of the potential gravity of the symptoms, prenatal diagnosis is essential. Therefore, fetal ultrasound combined with fetal MRI is important. The lesions are usually seen with the ultrasound in the late second and third semester of gestation [8,9]. The diagnosis is suspected in front of an elevated rate of alpha-fetoprotein, associated with a facial mass and polyhydramnios [9,10]. The prenatal MRI evaluates invasion of the brain, fetal airways, and precises the limits of the mass and its contact with the trachea and the umbilical cord [3,9,11]. All those elements allow to plan a tracheostomy (perinatal tracheostomy), or an EXIT (ex utero intrapartum treatment). CT-scan is not usually performed. This exam allows to eliminate bone involvement [3].

There is three surgical management possible for congenital teratomas: in utero procedure can be used for giant teratomas, on placenta support during EXIT intervention or postnatal.

EXIT is indicated when the lesion completely obstructs the airway tract, and during the procedure the neonate can be intubated or if it's not possible a tracheostomy is performed. Then EXIT intervention provides a safe control of the airway by removing the tumor, during placental support. This approach requires a multidisciplinary team involving ENT surgeon, pediatric surgeon, obstetrics, anesthesia and neonatology [12,13].

The prognosis of oropharyngeal teratoma is poor because of the airways obstruction [14]. It depends on the volume of the lesion and the involvement of essential structures.

## 4 Conclusion

Epignathi are not a common tumor. It should be diagnosed as early as possible. The treatment is strictly surgical. Earlier the lesion is highlighted more adapted will be the surgical management and complications as respiratory distress avoided.

## Consent

Written informed consent was obtained from the patient's parents/legal guardian for publication and any accompanying images. A copy of the written consent is available for review by the Editor-in-Chief of this journal on request.

## Ethical approval

This study is exempt from ethical approval at our institution (Habib Thameur Hospital, Tunis, Tunisia).

## Funding

No fundings.

## Author contribution

Dorra Chiboub: Writing - supervision.

Nadia Romdhane: Supervision.

Selima Jouini: Writing - Original draft.

Maya Cherif: Writing -Data collection.

Achraf Chedly: Supervision.

Chiraz Mbarek: Supervision.

## Guarantor

Selima Jouini.

## Conflict of interest statement

No conflicts of interest.
